# Prevalence and Risk Factors of Cardiovascular Autonomic Neuropathy in Individuals with Type 1 Diabetes Mellitus: A Systematic Review and Meta-Analysis

**DOI:** 10.31083/j.rcm2507244

**Published:** 2024-07-03

**Authors:** Xin Huang, Yun Bao, Jie Wang, Limin Tian

**Affiliations:** ^1^School of Clinical Medicine, Ningxia Medical University, 750000 Yinchuan, Ningxia, China; ^2^Department of Endocrinology, Gansu Provincial Hospital, 730000 Lanzhou, Gansu, China; ^3^Gansu Province Metabolic Disease Clinical Research Center, 730000 Lanzhou, Gansu, China; ^4^Institute of Clinical Research and Evidence Based Medicine, Gansu Provincial Hospital, 730000 Lanzhou, Gansu, China

**Keywords:** cardiac autonomic neuropathy, type 1 diabetes mellitus, prevalence, meta-analysis

## Abstract

**Background:**

Cardiac autonomic neuropathy (CAN) is a severe complication of type 1 diabetes mellitus (T1DM). This meta-analysis aimed to synthesize relevant literature on the prevalence of CAN and its risk factors in individuals with T1DM.

**Methods:**

We screened relevant literature from databases based on predefined search criteria until June 28, 2022. Data extraction and quality assessment were conducted independently by two reviewers. A meta-analysis was conducted to determine the prevalence of CAN and its risk factors in individuals with T1DM using a random-effects model. A subgroup analysis was conducted to assess variations in CAN prevalence based on diabetes duration, diagnostic criteria, study quality, study design, and geographic region of the participants.

**Results:**

A total of 21 studies provided information on the prevalence of CAN, while 18 studies explored the potential risk factors for CAN. The overall estimated prevalence of CAN in individuals with T1DM was 25.8% (95% confidence interval (95% CI): 0.208–0.307), with no significant differences observed among the five regions. Additionally, smoking, lipid abnormalities, hypertension, duration of diabetes, increased body mass index, elevated glycated haemoglobin concentrations, and presence of chronic complications of diabetes, such as diabetic retinopathy, diabetic neuropathy, and diabetic nephropathy, were associated with a higher prevalence of CAN in individuals with diabetes.

**Conclusions:**

CAN is prevalent in individuals with T1DM worldwide. Efforts should be made to improve early screening and intervention for CAN, as well as to implement strategies aimed at improving or controlling early risk factors associated with CAN.

## 1. Introduction

Type 1 diabetes mellitus (T1DM) is characterized by the destruction of insulin-producing beta cells in the pancreas. This leads to insufficient insulin production resulting in elevated blood sugar levels, ketoacidosis, and potentially fatal outcomes [1]. In 2021, there were about 8.4 million individuals worldwide with type 1 diabetes. According to projections, the number of people with the disease will increase to 13.5–17.4 million by 2040 (60–107% higher than in 2021) [2].

Cardiac autonomic neuropathy (CAN) arises from the impairment of sympathetic and parasympathetic nerve fibers that innervate the heart and blood vessels. This results in disturbances in cardiovascular autonomic regulation [3]. CAN poses a significant risk of myocardial dysfunction, arrhythmias, myocardial ischemia, chronic kidney disease, and sudden death. Owing to difficulties in diagnostic testing and the silent nature of early clinical symptoms, CAN is often detected at late stages, with a 5-year mortality rate as high as 50% [4]. CAN is a severe complication of T1DM that results from autonomic neuropathy and cardiovascular neural imbalance caused by diabetes. The prevalence of CAN in patients with T1DM ranges from 2–91% [5], and the wide variation in prevalence may be due to inconsistencies in the criteria used to diagnose CAN as well as significant variations in the study populations, particularly concerning CAN risk factors (such as age, gender, and duration of diabetes). Given the dangers of CAN and the uncertainty of its prevalence, we need to perform a meta-analysis to clarify the situation.

With the increasing prevalence of T1DM, the burden of CAN is expected to rise. Hence, it is imperative to identify factors that contribute to the prevalence of CAN in individuals with T1DM. This knowledge will greatly aid in the prevention and management of CAN. In addition to elevated blood sugar levels, several other important risk factors for CAN include age, duration of diabetes, presence of other microvascular complications, lipid profile, blood pressure, smoking habits, and body mass index (BMI) [6].

To date, comprehensive systematic reviews and meta-analyses examining the prevalence of CAN among individuals with T1DM are lacking. Therefore, this study aimed to address this gap by conducting a thorough literature search and meta-analysis to investigate the prevalence of CAN in T1DM patients. Furthermore, we aimed to identify and analyse risk factors associated with CAN in patients with T1DM.

## 2. Materials and Methods

### 2.1 Data Sources and Study Selection

This systematic review and meta-analysis adhered to the Preferred Reporting Items for Systematic Reviews and Meta-Analyses (PRISMA) statement [7] and was registered in the International Prospective Register of Systematic Reviews (PROSPERO) database (CRD42022345206). We searched the Cochrane Central Register of Controlled Trials, PubMed, Embase, and Web of Science databases for relevant articles published from their inception until June 28, 2022. The language used in this study was English. The search strategies were performed through a combination of Mesh terms and entry term: (“diabetes Mellitus, Type 1” or “type 1 diabetes” or “insulin dependent diabetes mellitus” or “diabetic ketoacidosis” or “T1DM”) and (“cardiovascular autonomic neuropathy” or “autonomic nervous system” or “cardiovascular system”).

### 2.2 Inclusion and Exclusion Criteria

The inclusion criteria were as follows: (1) studies on individuals with type 1 diabetes; (2) studies reporting accurate diagnostic criteria for CAN; (3) the prevalence or risk factors for CAN in individuals with type 1 diabetes; (4) CAN risk factor outcomes in T1DM patients reported as odds ratio (OR) (or calculable); (5) observational studies, including case-control, cross-sectional, and cohort studies. The exclusion criteria were as follows: (1) studies containing incomplete data; (2) studies in languages other than English; (3) studies with a sample size of less than 50; and (4) reviews, conference abstracts, case reports, editorials, and commentaries.

### 2.3 Data Extraction and Risk of Bias Assessment

After removing duplicate studies, two researchers conducted a thorough evaluation of eligible publications, adhering to the predetermined inclusion and exclusion criteria. Assessments were performed based on the titles and abstracts of publications. If at least one reviewer determined that the abstract met the inclusion criteria, the full-text articles were procured. Two reviewers independently assessed each publication to ascertain whether it was suitable for final inclusion in the study; any disparities were resolved through discussions. Two reviewers extracted information, including the first author’s name, publication year, study location, sample size, duration of diabetes, diagnostic criteria, the prevalence of CAN in T1DM, and reported risk factors, among others, were recorded and stored in Excel (version 2021, Microsoft Corporation, Redmond, WA, USA) for further analysis. The risk of bias in the cohort and case-control studies was independently evaluated by two researchers using the Newcastle-Ottawa Scale (NOS) [8]. NOS evaluates the quality of the literature using the semi-quantitative principle of the star rating system, evaluating three aspects selection, comparability, and exposure/outcome. Selection is divided into 4 entries, comparability is a separate entry, and exposure/outcome is divided into 3 entries. Except for comparability, which has a maximum of 2 stars, the selection and exposure/outcome have a maximum of 1 star per item (out of 9 stars), with higher scores suggesting higher study quality. They also evaluated the quality of the included cross-sectional studies based on five dimensions–sample representativeness, sample size, participation rate, outcome assessment, and analytical methods [9], following the Strengthening the Reporting of Observational Studies in Epidemiology (STROBE) reporting guidelines [10]. Each article was assigned a total score ranging from 0 to 10, representing the overall risk of bias. Scores lower than seven were considered low quality, whereas scores of seven or higher indicated high quality. In the case of discrepancies, a third researcher made the final decision.

### 2.4 Summary of the Prevalence of CAN in Individuals with T1DM

Each study reported the prevalence of CAN in individuals with T1DM as a percentage (%). A random-effects model was used to calculate the overall prevalence of CAN in individuals with T1DM, with a 95% confidence interval (95% CI). The literature was divided into subgroups based on the diagnostic criteria, study quality, study design, duration of diabetes, and geographical region of the participants (i.e., the Americas, Europe, Asia, Africa, and Oceania). Prevalence data were analysed using RStudio (version 4.2.2, J.J. Allaire Corporation, Newton, MA, USA).

### 2.5 Meta-Analysis of Risk Factors for CAN in Individuals with T1DM

Random-effects meta-analysis and inverse variance methods were used to assess pooled ORs and 95% confidence intervals. When extracting effect estimates (i.e., ORs) solely from the studies, a natural logarithm transformation was applied to the data. A meta-analysis was conducted using Review Manager (version 5.4, Nordic Cochrane Center, Northern Europe) when at least two studies examined the same risk factors for CAN. Additionally, subgroup, sensitivity, and descriptive analyses were employed to explore clinical heterogeneity among studies. *$I^{2}$* statistics (no: 0–25%; low: 25–50%; moderate: 50–75%; high: 75–100%) and forest plots were used to assess heterogeneity. Sensitivity analysis was performed by sequentially excluding each study to determine the individual impact on the overall results. The Egger’s test was used to evaluate publication bias. A two-tailed *p*-value ≤ 0.05 was considered statistically significant.

### 2.6 Diagnostic Methods for CAN

Measurement of cardiovascular reflex tests (CARTs): according to Ewing’s protocol [11], patients were subjected to serial blood pressure and electrocardiogram tests, and CAN was assessed by four standard CARTs: heart rate responses to deep breathing (expiration: inspiration [E:I] ratio), to standing (30:15 ratio), and to the Valsalva maneuver to assess parasympathetic function and blood pressure responses to standing to assess sympathetic function. Orthostatic hypotension was defined as a reduction of systolic blood pressure of at least 20 mmHg or diastolic blood pressure of at least 10 mmHg within three minutes of standing up [4]. CAN was defined as at least two abnormal results in three parasympathetic tests or the presence of orthostatic hypotension [12].

Measurement of heart rate variability (HRV): HRV refers to variations between consecutive heartbeats and cardiac cycles under the control of the autonomic nervous system. While lying on their back in a quiet room, participants underwent 10 minutes of continuous electrocardiograms to assess heart rate variability. Seven HRV parameters were evaluated across three domains: (i) time-domain measuring the overall HRV and including the standard deviation and root-mean-squared difference of successive normal-to-normal intervals, and heart rate; (ii) geometric domain measuring the Triangular index, another measure of overall HRV, whereby the total number of all RR intervals (duration of the interval between two R waves on the electrocardiogram) divided by the height of the histogram of all RR intervals measured on a discrete scale with bins of 7.8125 ms (1/128 s), and (iii) frequency-domain measuring low frequency (LF), defined as >0.04 and <0.15 Hz, high frequency (HF), defined as >0.15 and <0.4 Hz, and LF:HF, which represents the balance between the sympathetic and parasympathetic branches [13]. CAN was defined as ≥2 HRV abnormalities (out of 7).

## 3. Results

### 3.1 Literature Retrieval

**Fig. 1.**
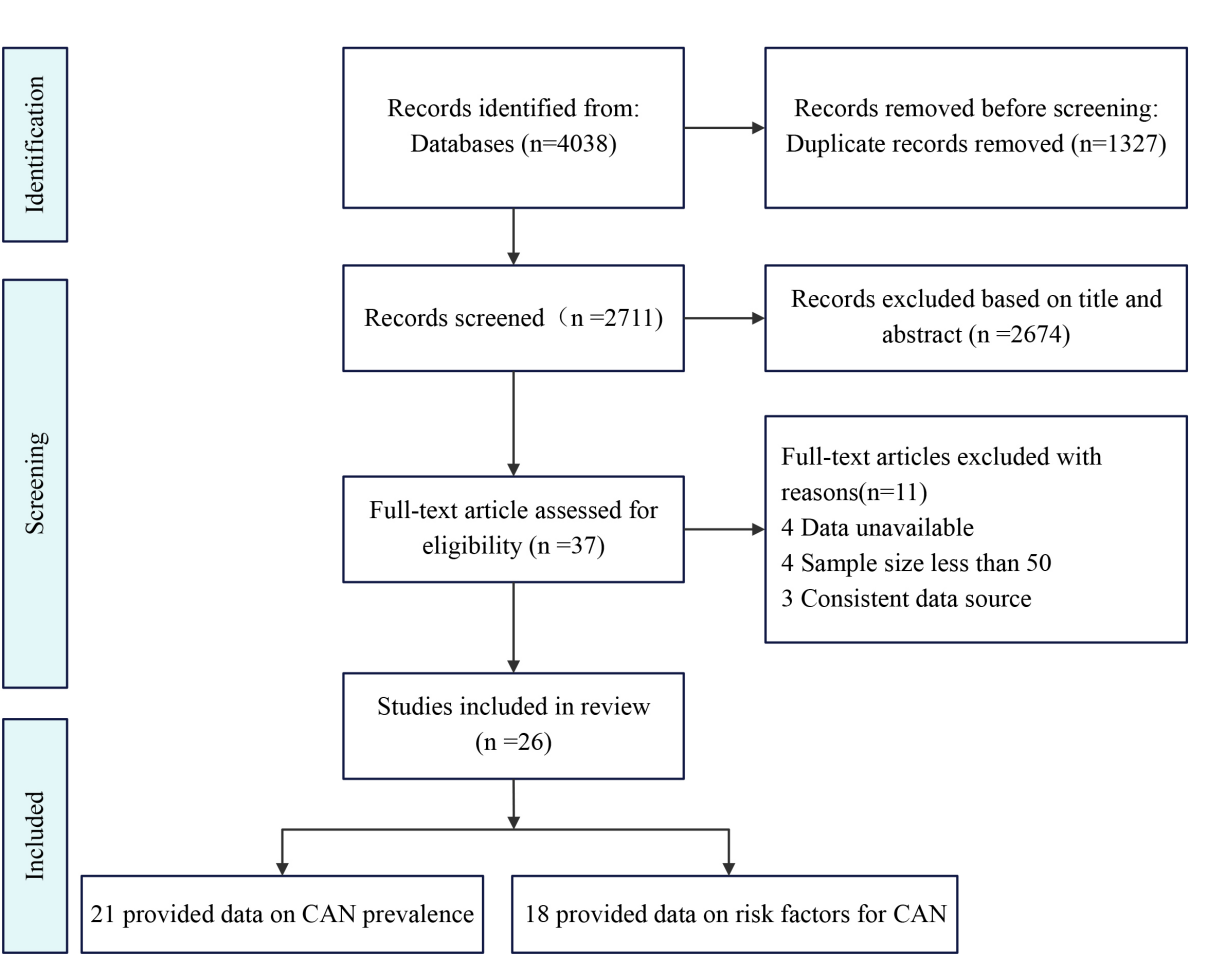
**Preferred Reporting Items for Systematic Reviews and Meta-Analyses (PRISMA) flow diagram of this systematic review and meta-analysis. **CAN, cardiac autonomic neuropathy.

We initially retrieved 4038 articles through keyword searches, of which 1327 were identified as duplicates and subsequently removed. Following the screening of titles and abstracts and the evaluation of whether the articles met the inclusion and exclusion criteria, we obtained 37 articles. Further examination of these articles revealed that four had sample sizes less than 50, four lacked or had unavailable data, and three articles had data derived from the same included literature. Finally, 26 studies were included in the meta-analysis [13, 14, 15, 16, 17, 18, 19, 20, 21, 22, 23, 24, 25, 26, 27, 28, 29, 30, 31, 32, 33, 34, 35, 36, 37, 38]. The article selection process was summarized according to PRISMA guidelines (Fig. 1). Characteristics A total of 26 studies met the inclusion criteria, including a cohort of 9414 individuals with CAN. The individuals were from Italy, Brazil, Denmark, Canada, Egypt, Pakistan, Australia, the United States, Bulgaria, France, the United Kingdom, China, South Korea, Greece, Lithuania, and Poland (Table 1, Ref. [13, 14, 15, 16, 17, 18, 19, 20, 21, 22, 23, 24, 25, 26, 27, 28, 29, 30, 31, 32, 33, 34, 35, 36, 37, 38]). All studies had a clear sex distribution, with a man-to-woman ratio of 99:100 (4678 *vs.*4736). Seven studies included children [13, 14, 15, 16, 17, 18, 19], whereas the remaining studies included only adults. The majority of individuals had a duration of diabetes ranging from 5 to 10 years or from 10 to 20 years, with only four studies reporting over 20 years [20, 21, 22, 23]. Furthermore, most studies diagnosed CAN using the CARTs, whereas two studies used HRV [14, 15, 16, 17]. Studies on CAN were conducted based on surveys in healthcare centers covering geographical regions across all five continents, with over one-third of the surveys conducted in Europe (42%, n = 11) and only one study from Oceania [17].

**Table 1.** **Characteristics of the included studies**.

Study	Country	Study design	Age, years #	Sample size (M/F)	Diabetes duration #	Method for diagnosing CAN	Prevalence (%) CAN	Risk factors assessed	Source of study population
Hansen *et al*., 2022 [22]	Denmark	CSS	55.0 ± 10.4	258 (121:137)	39.00 ± 10.35	CARTs	34.0%	Sex, smoking	Steno Diabetes Center Copenhagen (SDCC) in Denmar
Serdarova *et al*., 2021 [30]	Bulgaria	CSS	38.9 ± 12.3	235 (97:138)	12.00 ± 12.68	CARTs	23.0%	Dyslipidemia, somking, diabetic retinopathy	Department of Endocrinology, Medical University of Sofifia, Bulgaria
Riguetto *et al*., 2019 [31]	Brazil	CSS	32.0 ± 12.6	102 (35:67)	15.00 ± 9.02	CARTs	38.0%	Dyslipidemia, hypertension, sex, diabetic retinopathy/neuropathy/nephropathy	University of Campinas and University of São Paulo
Pawliński *et al*., 2019 [24]	Poland	CCS	31.0 ± 12.0	93 (33:60)	22.00 ± 29.37	CARTs	None	Age, diabetes duration, HbA1c, diabetic nephropathy	Local clinical centre
Pan *et al*., 2019 [25]	China	CCS	53.6 ± 13.1	73 (39:34)	9.00 ± 9.44	CARTs	None	Age, diabetes duration, HbA1c, sex, diabetic neuropathy, BMI	13 hospitals in Beijing
Kim *et al*., 2019 [32]	Korea	CSS	22.2 ± 2.9	95 (45:50)	13.10 ± 4.80	CARTs	12.6%	Sex	Outpatient clinic at Seoul National University Children’s Hospital
Jun *et al*., 2019 [33]	Korea	CSS	39.9 ± 14.0	80 (35:45)	10.10 ± 7.30	CARTs	45.0%	Sex, smoking	Samsung Medical Center
Admoni *et al*., 2019 [23]	Brazil	CSS	34.0 ± 12.6	341 (131:210)	21.00 ± 8.93	CARTs	29.0%	Dyslipidemia, hypertension, sex, smoking, diabetic retinopathy/neuropathy	Faculdade de Medicina, Universidade de São Paulo
Metwalley *et al*., 2018 [18]	Egypt	CSS	15.1 ± 3.3	60 (21:39)	7.95 ± 3.83	CARTs	36.7%	Diabetes duration, dyslipidemia, sex, hypertension, diabetic retinopathy/nephropathy	Outpatient pediatric diabetes clinic of Children’s Hospital, Assiut University
Matta *et al*., 2018 [20]	France	CS	50.0 ± 11.0	175 (99:76)	26.00 ± 11.00	CARTs	34.3%	Diabetes duration, dyslipidemia, hypertension, sex, BMI, smoking, diabetic retinopathy/neuropathy/nephropathy	Department of Diabetes and Metabolism of Toulouse University Hospital
Jaiswal *et al*., 2018 [14]	America	CS	18.0 ± 4.0	1646 (821:825)	7.80 ± 1.81	HRV	12.3%	Diabetes duration, HbA1c, sex, smoking	Search for Diabetes in Youth Registry Study
Razanskaite *et al*., 2017 [13]	Lithuania	CCS	19.2 ± 4.3	208 (89:119)	14.00 ± 4.36	CARTs	None	Sex	Swiss project “Genetic Diabetes in Lithuania”
Tannus *et al*., 2014 [34]	Brazil	CSS	33.4 ± 13.0	151 (70:81)	16.30 ± 9.50	CARTs	30.5%	Diabetes duration, dyslipidemia, hypertension, sex, smoking, diabetic retinopathy/neuropathy/nephropathy	Diabetes unit of State University of Rio de Janeiro
Mogensen *et al*., 2012 [26]	Denmark	CCS	54.8 ± 7.0	56 (33:23)	35.00 ± 10.36	CARTs	None	Hypertension, sex, smoking, diabetic retinopathy	Outpatient clinic of type 1 diabetic patients at Steno Diabetes Center and the Diabetes Unit, Rigshospitalet
Voulgari *et al*., 2011 [35]	Greece	CSS	34.8 ± 10.1	200 (110:90)	18.00 ± 26.14	CARTs	42.0%	Age, diabetes duration, HbA1c, hypertension, sex, smoking, diabetic retinopathy/neuropathy	Laiko General Hospital
Pavy-Le *et al*., 2010 [27]	France	CCS	47.0 ± 12.0	684 (350:334)	22.00 ± 11.00	CARTs	None	Diabetic retinopathy/neuropathy/nephropathy	University of Toulouse
Witte *et al*., 2005 [28]	England	CS	31.3 ± 8.9	956 (515:441)	13.50 ± 8.30	CARTs	17.0%	Age, diabetes duration, hypertension, sex, smoking, diabetic retinopathy, BMI, HbA1c	31 European diabetic clinic populations
Stella *et al*., 2000 [21]	America	CS	28.6 ± 7.8	373 (203:170)	20.00 ± 7.37	CARTs	28.0%	Hypertension, sex, smoking, diabetic retinopathy/neuropathy/nephropathy	Pittsburgh Epidemiology of Diabetes Complications Study
Maddaloni *et al*., 2021 [36]	Italy	CSS	37.0 ± 13.3	80 (44:36)	17.00 ± 12.83	CARTs	26.0%	None	Diabetes Units of Sapienza University and of Campus Bio-Medico University
Gomes *et al*., 2021 [19]	Brazil	CSS	16.4 ± 1.9	328 (144:184)	8.10 ± 4.30	CARTs	12.5%	None	14 public clinics, in ten Brazilian cities, from four geographic regions
Christensen *et al*., 2018 [37]	Denmark	CSS	22.0 ± 1.6	156 (65:91)	11.30 ± 5.10	CARTs	9.0%	None	Outpatient clinic at Steno Diabetes Center Copenhagen, Gentofte, Denmark
Orlov *et al*., 2015 [16]	Canada	CS	30.5 ± 7.0	370 (182:188)	16.00 ± 8.23	CARTs	12.7%	None	Joslin Diabetes Center
Abd *et al*., 2011 [15]	Egypt	CS	12.3 ± 4.1	57 (27:30)	5.90 ± 3.70	CARTs	14.0%	None	Endocrine clinic in the National Research Centre
Khoharo *et al*., 2009 [38]	Pakistan	CSS	35.2 ± 10.6	50 (32:18)	13.00 ± 7.30	CARTs	20.0%	None	Department of Medicine, Liaquat University Hospital, Hyderabad/Jamshoro
Varley *et al*., 2022 [17]	Australia	CS	16.5 ± 2.3	1153 (576:577)	8.00 ± 4.23	HRV	27.0%	None	Clinical registry of the Diabetes Complications Assessment Service at the Children’s Hospital in Sydney
Braffett *et al*., 2020 [29]	America	CS	27.0 ± 7.4	1434 (761:673)	5.00 ± 5.20	CARTs	44.0%	None	The Diabetes Control and Complications Trial/Epidemiology of Diabetes Interventions and Complications (DCCT/EDIC) Research Group

M, male; F, female; CAN, cardiac autonomic neuropathy; CS, cohort study; CSS, cross-sectional study; CCS, case control study; CARTs, cardiac autonomic reflex test; HRV, heart rate variability; BMI, body mass index; HbA1c, hemoglobin A1c; #, mean ± standard deviation (SD).

### 3.2 Quality Assessment

The twenty-six included studies were generally of good quality. For five case-control studies [13, 24, 25, 26, 27], of which two studies had scores below 7 (6 and 5 points) on NOS [13, 24]. All cohort studies (eight studies) had scores greater than 7 on NOS [14, 15, 16, 17, 20, 21, 28, 29] (Table 2, Ref. [13, 14, 15, 16, 17, 20, 21, 24, 25, 26, 27, 28, 29]). In addition, among the 13 cross-sectional studies [18, 19, 22, 23, 30, 31, 32, 33, 34, 35, 36, 37, 38], three had scores below 7 [22, 33, 38] (Table 3, Ref. [18, 19, 22, 23, 30, 31, 32, 33, 34, 35, 36, 37, 38]).

**Table 2.** **Newcastle-Ottawa quality assessment scale (NOS) for the eligible cohort, or case-control studies**.

Author (year)	Selection	Comparability	Exposure/outcome	Score
Pawliński *et al*., 2019 [24]	★★	★★	★★	★★★★★★
Pan *et al*., 2019 [25]	★★★	★★	★★	★★★★★★★
Razanskaite *et al*., 2017 [13]	★★	/	★★★	★★★★★
Mogensen *et al*., 2012 [26]	★★	★★	★★★	★★★★★★★
Pavy *et al*., 2010 [27]	★★★	★★	★★	★★★★★★★
Varley *et al*., 2022 [17]	★★★	★★	★★	★★★★★★★
Braffett *et al*., 2020 [29]	★★★	★★	★★★	★★★★★★★★
Matta *et al*., 2018 [20]	★★★	★★	★★★	★★★★★★★★
Jaiswal *et al*., 2018 [14]	★★★	★★	★★	★★★★★★★
Witte *et al*., 2005 [28]	★★★★	★★	★★	★★★★★★★★
Stella *et al*., 2000 [21]	★★★★	★★	★★	★★★★★★★★
Orlov *et al*., 2015 [16]	★★★	★★	★★	★★★★★★★
Abd *et al*., 2011 [15]	★★★	★	★★★	★★★★★★★

★: a star indicates that the criteria for each assessment entry are met. Except for comparability, which has a maximum of 2 stars, the selection and exposure/outcome have a maximum of 1 star per item (out of 9 stars).

**Table 3.** **Quality scoring of cross-sectional studies according to Strengthening the Reporting of Observational Studies in Epidemiology (STROBE) reporting guideline**.

Author (year)	Sample population	Sample size	Participation rate	0utcome assessment	Analytical methods	Score
Hansen *et al*., 2022 [22]	2	1	0	1	2	6
Serdarova *et al*., 2021 [30]	2	1	0	2	2	7
Riguetto *et al*., 2019 [31]	2	1	0	2	2	7
Kim *et al*., 2019 [32]	2	1	2	2	2	9
Jun *et al*., 2019 [33]	2	1	0	1	2	6
Admoni *et al*., 2019 [23]	2	1	0	2	2	7
Metwalley *et al*., 2018 [18]	2	1	0	2	2	7
Tannus *et al*., 2014 [34]	2	1	0	2	2	7
Voulgari *et al*., 2011 [35]	2	1	0	2	2	7
Maddaloni *et al*., 2021 [36]	2	1	0	2	2	7
Gomes *et al*., 2021 [19]	2	2	0	2	2	8
Christensen *et al*., 2018 [37]	2	1	2	2	2	9
Khoharo *et al*., 2009 [38]	1	1	0	2	1	5

### 3.3 Prevalence Analysis

**Fig. 2.**
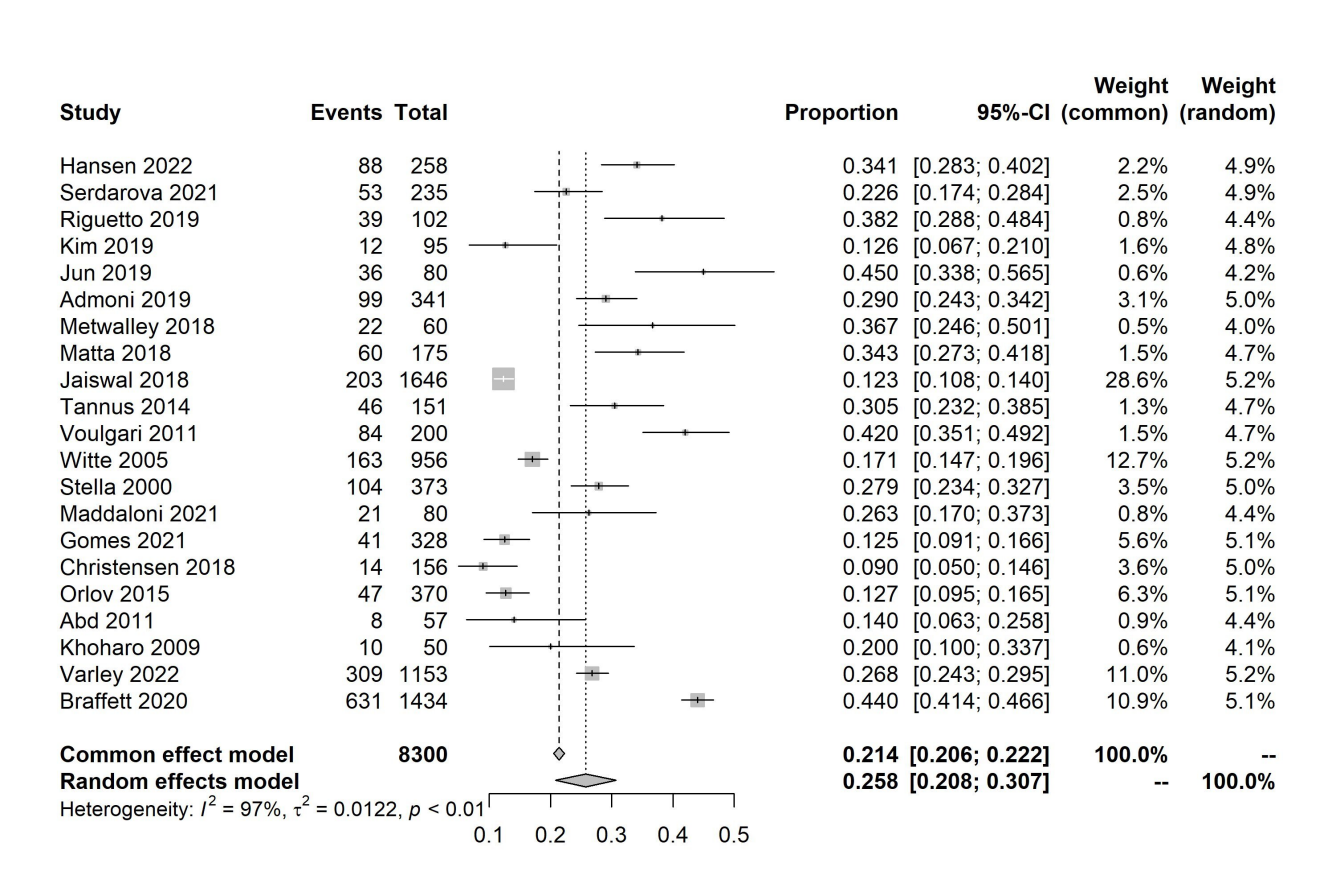
**Forest plot of meta-analysis of prevalence of cardiac autonomic neuropathy (CAN) in patients with type 1 diabetes mellitus (T1DM). **95% CI, 95% confidence interval.

A total of 21 studies were included in the meta-analysis. The prevalence of CAN according to the studies ranged from 9% to 45% (Fig. 2) [14, 15, 16, 17, 18, 19, 20, 21, 22, 23, 28, 29, 30, 31, 32, 33, 34, 35, 36, 37, 38]. Based on the random-effects model meta-analysis of all data points, the estimated overall prevalence of CAN was 25.8% (95% CI: 0.208–0.307, *$I^{2}$* = 97%, *p* < 0.01). According to the subgroup analysis, when categorizing the duration of diabetes into three groups: 5–10 years, 10–20 years, and >20 years, the prevalence of CAN in each group was 24%, 25%, and 31%, respectively. Among cohort studies, the estimated overall prevalence of CAN was 24%, whereas it was 27% in cross-sectional studies. The overall prevalence of CAN was higher in individuals diagnosed using CARTs than in those diagnosed using HRV. Finally, the estimated prevalence of CAN varies slightly across different regions, ranging from 25% to 27%. The comprehensive estimates obtained from the subgroup analyses are shown in Table 4 (detailed forest maps for each subgroup can be seen in **Supplementary Figs. 1–5**).

**Table 4.** **Results of subgroup analyses of prevalence by location, diagnostic criteria, study type, study quality, and diabetes duration**.

Subgroups		Number of included studies	Prevalence	95% CI	* $I^{2}$ *	*p* value
Location						
	Europe	7	26.2%	17.7–34.8%	95%	<0.01
	Americas	8	25.7%	17.1–34.3%	99%	<0.01
	Asia	3	25.6%	6.4–44.8%	92%	<0.01
	Africa	2	25.0%	2.8–47.1%	88%	<0.01
	Oceania	1	26.8%	24.3–29.5%	None	None
Diagnostic criteria						
	CARTs	19	26.5%	21.2–31.8%	96%	<0.01
	HRV	2	19.5%	5.4–33.7%	99%	<0.01
Study type						
	Cross-sectional study	13	27.1%	20.7–33.6%	93%	<0.01
	Cohort study	8	23.7%	15.6–31.8%	99%	<0.01
Study quality						
	Low risk	3	33.1%	19.7–46.5%	80%	<0.01
	High risk	18	24.6%	19.4–29.9%	97%	<0.01
Diabetes duration						
	5–10 years	6	24.2%	13.2–35.2%	99%	<0.01
	10–20 years	11	24.6%	17.3–31.9%	92%	<0.01
	>20 years	4	30.7%	27.5–33.9%	29%	0.24

CARTs, cardiac autonomic reflex test; HRV, heart rate variability; 95% CI, 95% confidence interval.

### 3.4 Analysis of Risk Factors

A meta-analysis of six studies showed that dyslipidemia [OR = 1.97, 95% CI: 1.34–2.90, *p* = 0.0005; *$I^{2}$* = 42%] was a risk factor for CAN in individuals with T1DM [18, 20, 23, 30, 31, 34]. Meta-analysis of nine studies indicated that hypertension [OR = 2.32, 95% CI: 1.73–3.11, *p* < 0.00001; *$I^{2}$* = 49%] was a risk factor for CAN in individuals with T1DM [18, 20, 21, 23, 26, 28, 31, 34, 35]. Eleven studies found that smoking was a risk factor for CAN in individuals with T1DM [OR = 1.16, 95% CI: 1.03–1.31, *p* = 0.01; *$I^{2}$* = 0%] [14, 20, 21, 22, 23, 26, 28, 30, 33, 34, 35]. In addition, three [20, 25, 28] and five studies [14, 24, 25, 28, 35] showed that BMI (per ${\mathrm{kg/m}}^{2}$ increase) [OR = 1.06, 95% CI: 1.00–1.12, *p* = 0.05; *$I^{2}$* = 0%] and HbA1c (per % increase) [OR = 1.23, 95% CI: 1.15–1.33, *p* < 0.00001; *$I^{2}$* = 0%] were significantly associated with an increased risk of CAN in individuals with T1DM, without significant heterogeneity. Furthermore, diabetic retinopathy, diabetic nephropathy [OR = 1.92, 95% CI: 1.07–3.43,* p* = 0.03; *$I^{2}$* = 66%], and diabetic neuropathy [OR = 2.95, 95% CI: 2.00–4.35, *p* < 0.00001; *$I^{2}$* = 53%] increased the risk of developing CAN in individuals with T1DM. However, the meta-analysis showed significant heterogeneity in diabetic retinopathy [OR = 2.02, 95% CI: 1.41–2.89, *p* = 0.0001; *$I^{2}$* = 82%] (Table 5).

**Table 5.** **Pooled risk factors of synthesized effect size of 11 risk factors for CAN in individuals with T1DM**.

No.	Risk factors	Number of included studies	OR	95% CI	Heterogeneity		*p *value of association
					* $I^{2}$ *	*p* value	
1	Age (per year increase)	4	1.06	(0.96, 1.17)	89%	<0.00001	0.28
2	BMI (per ${\mathrm{kg/m}}^{2}$ increase)	3	1.06	(1.00, 1.12)	0%	0.73	0.05
3	Diabetes duration (per year increase)	8	1.07	(0.99, 1.16)	30%	0.19	0.08
4	HbA1c (per % increase)	5	1.23	(1.15, 1.33)	0%	0.61	<0.00001
5	Dyslipidemia	6	1.97	(1.34, 2.90)	42%	0.13	0.0005
6	Hypertension	9	2.32	(1.73, 3.11)	49%	0.05	<0.00001
7	Sex	15	1.04	(0.88, 1.22)	44%	0.04	0.68
8	Smoking	11	1.16	(1.03, 1.31)	0%	0.97	0.01
9	Diabetic retinopathy	10	2.02	(1.41, 2.89)	82%	<0.00001	0.0001
10	Diabetic neuropathy	7	2.95	(2.00, 4.35)	53%	0.05	<0.00001
11	Diabetic nephropathy	6	1.92	(1.07, 3.43)	66%	0.01	0.03

BMI, body mass index; OR, odds ratio; 95% CI, 95% confidence interval; CAN, cardiac autonomic neuropathy; T1DM, type 1 diabetes mellitus; HbA1c, hemoglobin A1c.

It is worth noting that meta-analysis showed that gender [13, 14, 18, 20, 21, 22, 23, 25, 26, 28, 31, 32, 33, 34, 35] [OR = 1.04, 95% CI: 0.88–1.22, *p* = 0.68; *$I^{2}$* = 44%] and age [24, 25, 28, 35] [OR = 1.06, 95% CI: 0.96–1.17,* p *= 0.28; *$I^{2}$* = 89%] were not independent risk factors for CAN (detailed forest maps for each subgroup can be seen in **Supplementary Figs. 6–16**).

### 3.5 Sensitivity Analysis and Publication Bias

**Fig. 3.**
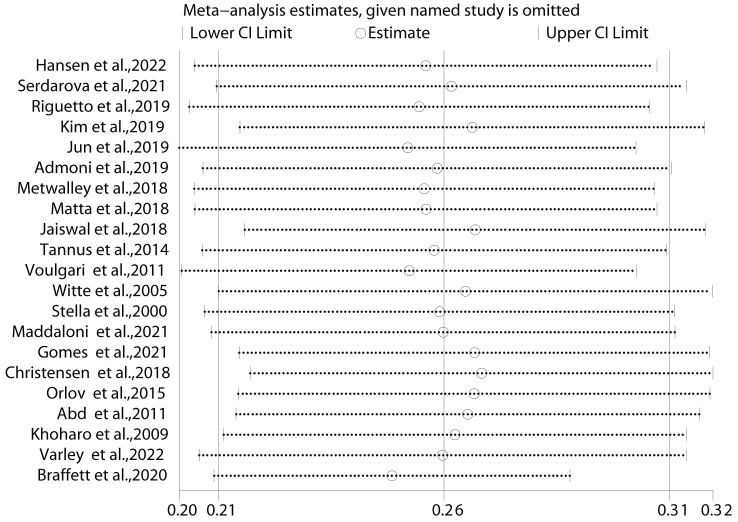
**Results of sensitivity analysis for prevalence-specific outcomes.** The effect size was expressed as OR and 95% CI for all studies. OR, odds ratio; 95% CI, 95% confidence interval.

**Fig. 4.**
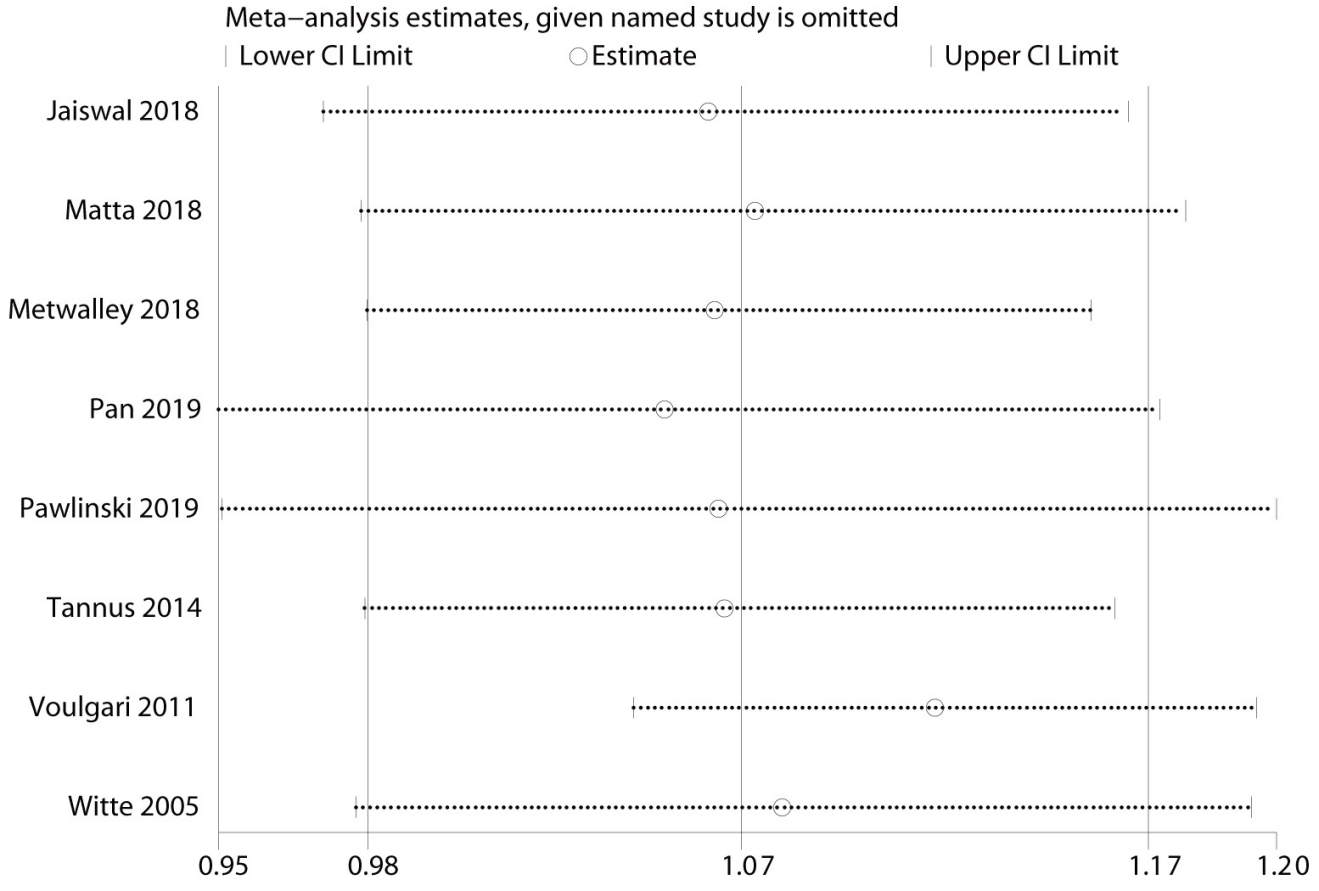
**Results of sensitivity analysis for duration of diabetes-specific outcomes. **The effect size was expressed as OR and 95% CI for all studies. OR, odds ratio; 95% CI, 95% confidence interval.

**Fig. 5.**
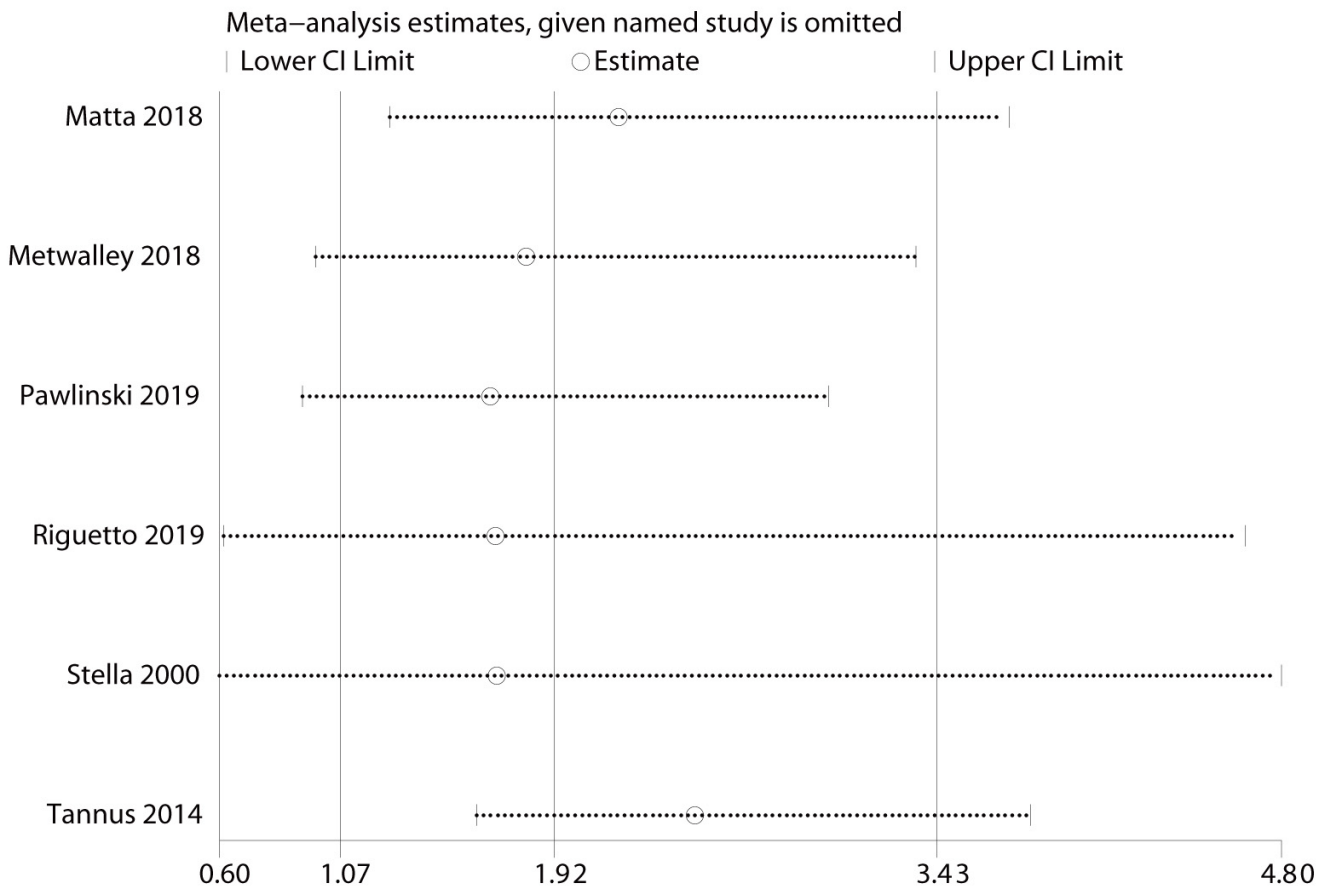
**Results of sensitivity analysis for diabetic nephropathy-specific outcomes.** The effect size was expressed as OR and 95% CI for all studies.

Sensitivity analysis showed that the analysis results for prevalence were stable (Fig. 3). After excluding the study by Voulgari *et al*. [35], the OR for duration of diabetes was 1.12 (95% CI: 1.05–1.19; *$I^{2}$* = 0%) (Fig. 4). For diabetic nephropathy, excluding four studies [18, 21, 24, 31], the combined results were not statistically significant (Fig. 5). Sensitivity analyses revealed that the other results were stable and consistent. Egger’s test was used to assess publication bias for each outcome, and the results showed no publication bias for any of the outcome measures (Table 6).

**Table 6.** **Egger’s test to assess the results of publication bias**.

No.	Index	*p*-value
1	Age (per year increase)	0.061
2	BMI (per ${\mathrm{kg/m}}^{2}$ increase)	0.120
3	Diabetes duration (per year increase)	0.230
4	HbA1c (per % increase)	0.296
5	Dyslipidemia	0.580
6	Hypertension	0.462
7	Sex	0.801
8	Smoking	0.259
9	Retinopathy	0.123
10	Diabetic neuropathy	0.836
11	Nephropathy	0.580
12	Prevalence	0.147

BMI, body mass index; HbA1c, hemoglobin A1c.

## 4. Discussion

This systematic meta-analysis reports, for the first time, an overall prevalence of 25.8% (95% CI: 0.208–0.307) for CAN in patients with T1DM. In addition, smoking, dyslipidemia, hypertension, duration of diabetes, higher BMI, increased HbA1c concentrations, diabetic retinopathy, diabetic neuropathy, and diabetic nephropathy have all been associated with CAN in patients with T1DM.

The results of the subgroup analysis indicated that the prevalence of CAN in patients with T1DM varied from 25% to 27% among the five regions studied, and no significant difference was observed. The Nordic region exhibited the highest prevalence of CAN among individuals with T1DM, particularly in Denmark, where the prevalence was approximately 30%. This observation suggests a potential correlation between the incidence of T1DM and the prevalence of CAN in this region [39]. The CARTs, proposed by Ewing and Clarke, are the gold standard for objectively assessing the parasympathetic and sympathetic branches of the autonomic nervous system. According to the subgroup analysis, CARTs showed a higher prevalence than HRV testing, possibly because HRV testing can help identify patients at risk for CAN through electrocardiography [5]. However, due to the limited number of studies on HRV detection, no conclusions could be drawn, and further exploration is needed in larger samples [40]. During subgroup analysis based on diabetes duration, we discovered a noteworthy trend: the prevalence of CAN in patients with T1DM increased significantly as the disease duration increased. This finding strongly suggests that a longer duration of diabetes is a crucial risk factor for the development of CAN. The pathogenesis of CAN is not fully understood, but recent research has found that its development is mainly associated with hyperglycaemic toxicity, which directly increases the production of reactive oxygen species (ROS) and advanced glycation end-products (AGEs). The accumulated AGEs inside and outside cells bind to AGE receptors, stimulate phosphoinositide 3-kinase and protein kinase B, activate nuclear factor-kappa B, and ultimately lead to neuronal damage [41]. As the disease progresses, the ability of patients with diabetes to regulate their blood sugar levels decreases, which consequently increases the severity of hyperglycaemic toxicity, making them more susceptible to autonomic neuropathy. According to CARTs, the prevalence of CAN increases by 4.6–6% per year with the increasing duration of diabetes [16, 41].

According to our meta-analysis, several risk factors were identified for the development of CAN in patients with T1DM. These factors include HbA1c levels, duration of diabetes, hypertension, dyslipidemia, obesity, smoking, and the presence of microvascular complications, such as neuropathy, nephropathy, and retinopathy. These findings are consistent with those of previous studies conducted on this subject [42, 43]. The present study demonstrated a statistically significant correlation between increased HbA1c levels and the incidence of CAN in individuals with T1DM. This finding is consistent with the results of numerous previous studies and pooled data, further supporting the association between elevated HbA1c levels and the development of CAN in this patient population [43, 44]. The primary explanation for this connection lies in the effect of hyperglycemia on the induction of oxidative stress and the formation of toxic AGEs. These biochemical changes can alter mitochondrial function, membrane permeability, and endothelial function, contributing to the development of CAN in individuals with T1DM [45, 46]. These diverse pathways trigger gene expression, transcription factor activation, and the disruption of various cellular functions. Moreover, they induce changes in cell-cell communication and interactions with the surrounding matrix. Collectively, these mechanisms contribute to neuronal dysfunction and eventual cell death [4, 10], thereby increasing the likelihood of developing CAN.

Several studies have consistently demonstrated a significant involvement of cardiovascular risk factors in the development of CAN [46, 47]. Risk factors include hypertension, BMI, dyslipidemia, and smoking. Our findings align closely with those of previous studies, further emphasizing the important roles of these factors in the occurrence and progression of CAN [46, 47]. Notably, these factors have at least two common points: they may be modifiable and are associated with insulin resistance, suggesting that CAN may occur concurrently with metabolic syndrome. The altered autonomic nervous function is often seen in cases of obesity [16, 17]; however, the mechanisms by which being overweight leads to autonomic dysfunction remain unclear. Several interrelated functional changes, such as inflammation [29], endothelial dysfunction [22], leptin dysfunction [30], and gastric hunger hormone regulation [31], may lead to increased sympathetic neural activity, decreased cardiac stress reflex sensitivity, and promote CAN development [32]. Diabetic nephropathy is also closely related to the development of CAN, as it may mediate changes in renal glomerular hemodynamics, blood pressure, and the diurnal rhythm of proteinuria [48].

Furthermore, distinct autonomic dysfunction states have been described in patients with CAN, including erythropoietin deficiency, anaemia, and early dysregulation of erythropoietin production. These findings highlight the additional factors that contribute to the development of autonomic dysfunction in individuals with CAN [33]. Anaemia is a predictive factor for the progression of kidney disease, whereas erythropoietin has a direct renal protective effect. Therefore, both anaemia and erythropoietin deficiency may contribute to diabetic kidney damage. Based on the data presented in Table 3, our findings align with those of the multicentre EURODIAB (EUROpe and DIABetes) Insulin-dependent Diabetes Mellitus (IDDM) Complications Study [49], indicating that there was no significant difference in the prevalence of CAN between male (35%) and female (37%) patients with T1DM. This suggests that sex is not a risk factor for the development of CAN, further indicating that our results are in line with those of previous research. In previous studies, increased age has been considered a risk factor for CAN [43, 50, 51]. However, our meta-analysis of risk factors of CAN showed that, contrary to the reports of previous studies, age was not a significant risk factor. Considering that these studies were based solely on four individual studies that evaluated the impact of age on CAN, further analysis is necessary as new data become available to update our understanding.

In addition, Pan *et al*. [25] found that in the baseline data of the two groups of T1DM patients diagnosed with CAN and non-CAN, the proportion of coronary heart disease complications was 4.4% versus 0%, *p* > 0.05, and the proportion of cerebral infarction complications was 2.2% versus 0%, *p* > 0.05. Stella *et al*. [21] demonstrated that macrovascular disease is a risk factor for the development of CAN in the T1DM population [risk ratio = 1.57, 95% CI: 1.01–2.44] (specific *p* value not extracted). In the study by Witte *et al*. [28], we learned that a history of CVD disease was a risk factor for the development of CAN [OR = 1.88, 95% CI: 1.02–3.47, *p* < 0.05].

This study has several limitations that should be acknowledged. First, all the studies included in this meta-analysis were observational, which may introduce potential biases and limit the generalizability of the findings. Second, this systematic review did not include non-English articles, which may have introduced language bias. The included studies predominantly came from Europe and may not be representative of regions such as the Eastern or Western Mediterranean, thus limiting the generalizability of the results to these areas. Third, certain unpublished articles and studies included in this analysis did not employ reliable and objective methods for diagnosing T1DM, nor did they provide clear diagnostic criteria. This may have introduced publication bias and affected the overall accuracy and reliability of the results. Fourth, relatively few articles on certain influencing factors were included in this study, making it difficult to determine the relationship between these factors and the occurrence of CAN in patients with T1DM. Fifth, unfortunately, we did not include data on macrovascular disease associated with CAN in our study and only described these data in the text. Due to data limitations, we need to interpret this information with caution, and more studies should be conducted subsequently to expand this data. Moreover, methodological variations among the included studies inevitably led to high levels of heterogeneity in the overall prevalence rates. Additionally, several studies had relatively small sample sizes. To address these limitations, future research should prioritize large-scale, multicentre epidemiological studies. Such studies would enable a deeper understanding of the risk factors associated with CAN in patients with T1DM.

## 5. Conclusions

The findings of this study suggest that CAN is prevalent worldwide, and efforts should be made to enhance early screening and intervention for CAN as well as to implement strategies aimed at improving or controlling the risk factors of CAN in the early stages. The results of this study should be updated in a timely and regular manner with the availability of new epidemiological investigations.

## Data Availability

The data used to support the findings of this study are included within the article.

## Abbreviations

T1DM, type 1 diabetes mellitus; CAN, cardiac autonomic neuropathy; CARTs, cardiac autonomic reflex test; HRV, heart rate variability; CI, confidence interval; *$I^{2}$*, I-squared; NOS, Newcastle-Ottawa Scale; BMI, body mass index; STROBE, Strengthening the Reporting of Observational Studies in Epidemiology.

## Supplementary Material

Supplementary material associated with this article can be found, in the online version, at https://doi.org/10.31083/j.rcm2507244.
