# Supplementary material for: Prevalence and Risk Factors of Cardiovascular Autonomic Neuropathy in Individuals with Type 1 Diabetes Mellitus: A Systematic Review and Meta-Analysis
Source: Rev Cardiovasc Med. 2024 Jul 3;25(7):244. doi: 10.31083/j.rcm2507244 (PMC11317325; doi:10.31083/j.rcm2507244)
Supplement: Supplementary file 1 [file 2153-8174-25-7-244-s1.zip › search strategy.docx]

| Search | Query | Item found |
| --- | --- | --- |
| #1 | ("diabetes mellitus, type 1"[MeSH Terms] OR “Diabetic Ketoacidosis” 0[MeSH Terms] OR “Diabetes Complications”[MeSH Terms] OR "insulin dependent diabetes mellitus"[Title/Abstract] OR "type 1 diabetes"[Title/Abstract] OR “IDDM” [Title/Abstract] OR “T1DM” [Title/Abstract] OR “T1D” [Title/Abstract]) | 232,953 |
| #2 | （“insulin*” [Title/Abstract] AND “depend*” [Title/Abstract]）OR (“diabet*”[Title/Abstract] AND “acidos*”[Title/Abstract]) OR (“juvenil*”[Title/Abstract] AND “diabet*”[Title/Abstract]) OR (“diabet*”[Title/Abstract] AND “child*”[Title/Abstract]) OR (“diabet*”[Title/Abstract] AND “keto*” [Title/Abstract]) | 122,284 |
| #3 | #1 OR #2 | 306,434 |
| #4 | (("cardiacs"[Title/Abstract] OR "cardiac"[Title/Abstract]) AND ("autonomic nervous system"[MeSH Terms] OR "autonomic"[Title/Abstract] OR "autonomics"[Title/Abstract]) AND ("neuropathies"[Title/Abstract] OR "neuropathy"[Title/Abstract])) OR ("cardiac autonomic neuropathy"[Title/Abstract]) | 1434 |
| #5 | (("cardiovascular system"[MeSH Terms] OR "cardiovascular"[Title/Abstract] OR "cardiovasculars"[Title/Abstract]) AND ("autonomic nervous system"[MeSH Terms] OR "autonomic"[Title/Abstract] OR "autonomics"[Title/Abstract]) AND ("neuropathies"[Title/Abstract] OR "neuropathy"[Title/Abstract])) OR ("Cardiovascular Autonomic Neuropathy"[Title/Abstract]) | 2157 |
| #6 | #4 OR #5 | 2802 |
| #7 | #3 AND #6 | 1715 |

PUBMED
