# Supplementary material for: Prevalence and Risk Factors of Cardiovascular Autonomic Neuropathy in Individuals with Type 1 Diabetes Mellitus: A Systematic Review and Meta-Analysis
Source: Rev Cardiovasc Med. 2024 Jul 3;25(7):244. doi: 10.31083/j.rcm2507244 (PMC11317325; doi:10.31083/j.rcm2507244)
Supplement: Supplementary file 1 [file 2153-8174-25-7-244-s1.zip › Supplementary Material(fig1-16).docx]

**Supplementary Material：**


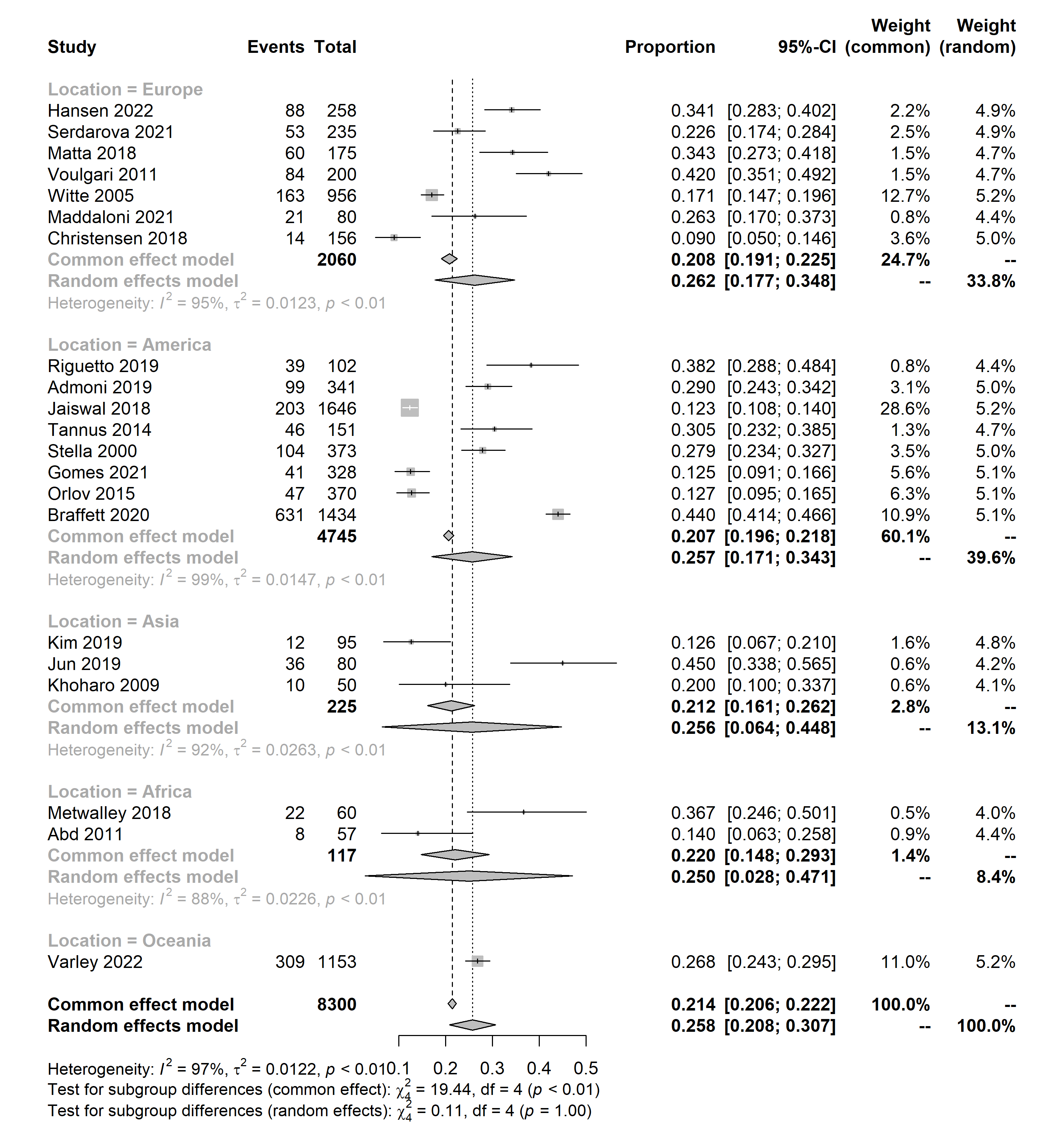


**Fig. 1. Subgroup analysis of prevalence according to geographic location.**


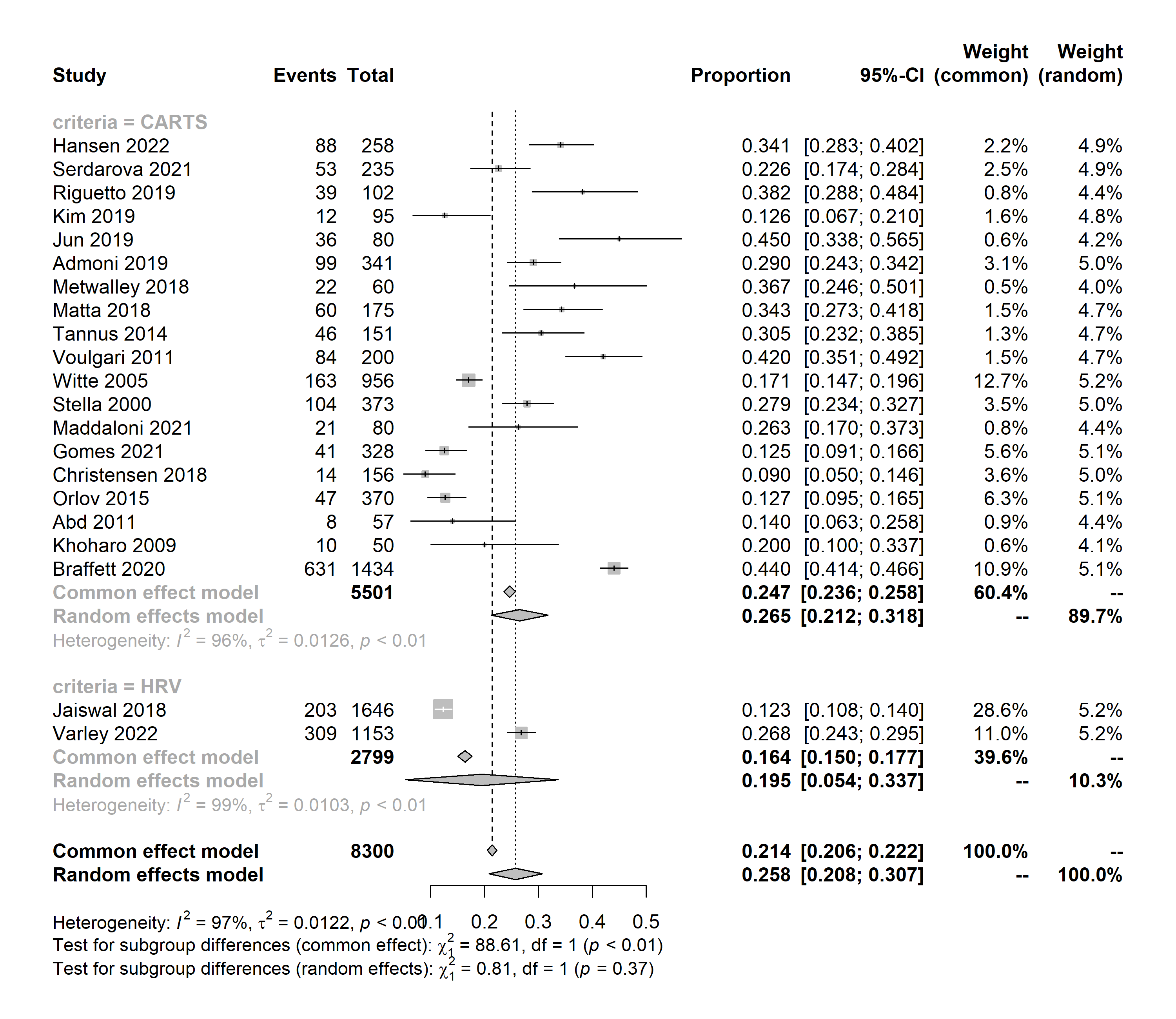


**Fig. 2. Subgroup analysis of prevalence according to diagnostic criteria.**


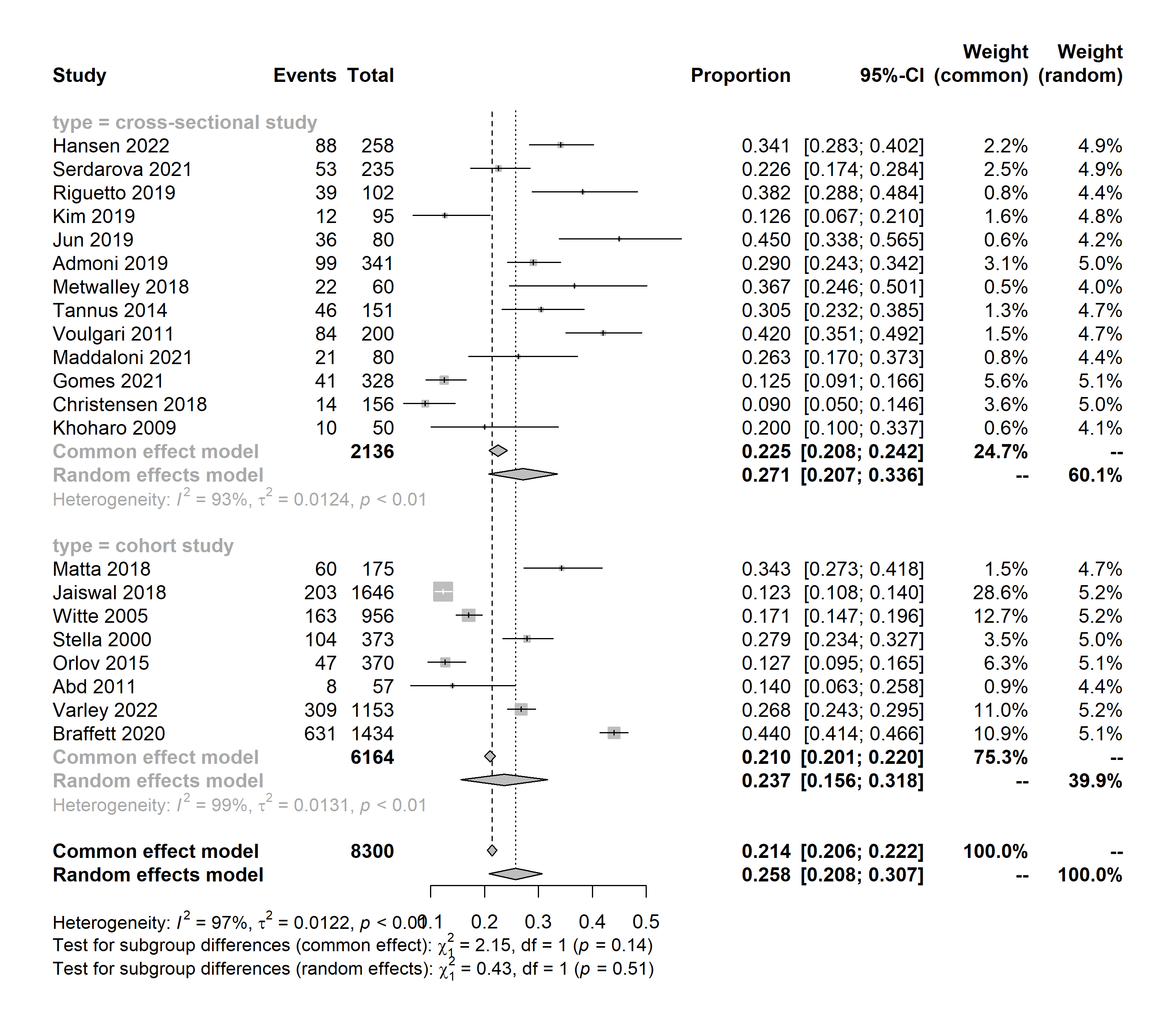


**Fig. 3. Subgroup analysis of prevalence according to** **study type.**


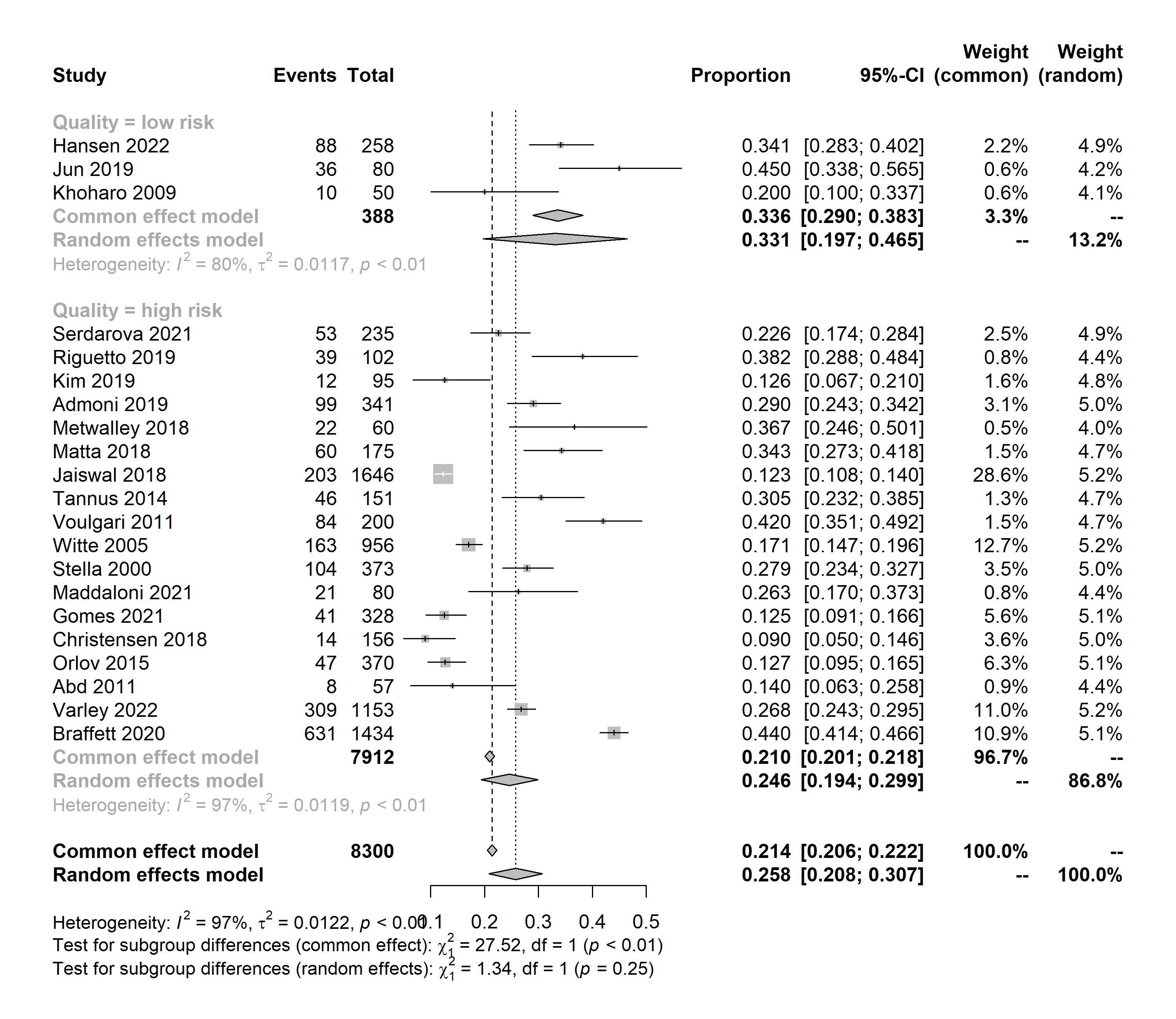


**Fig. 4. Subgroup analysis of prevalence according to** **study quality.**


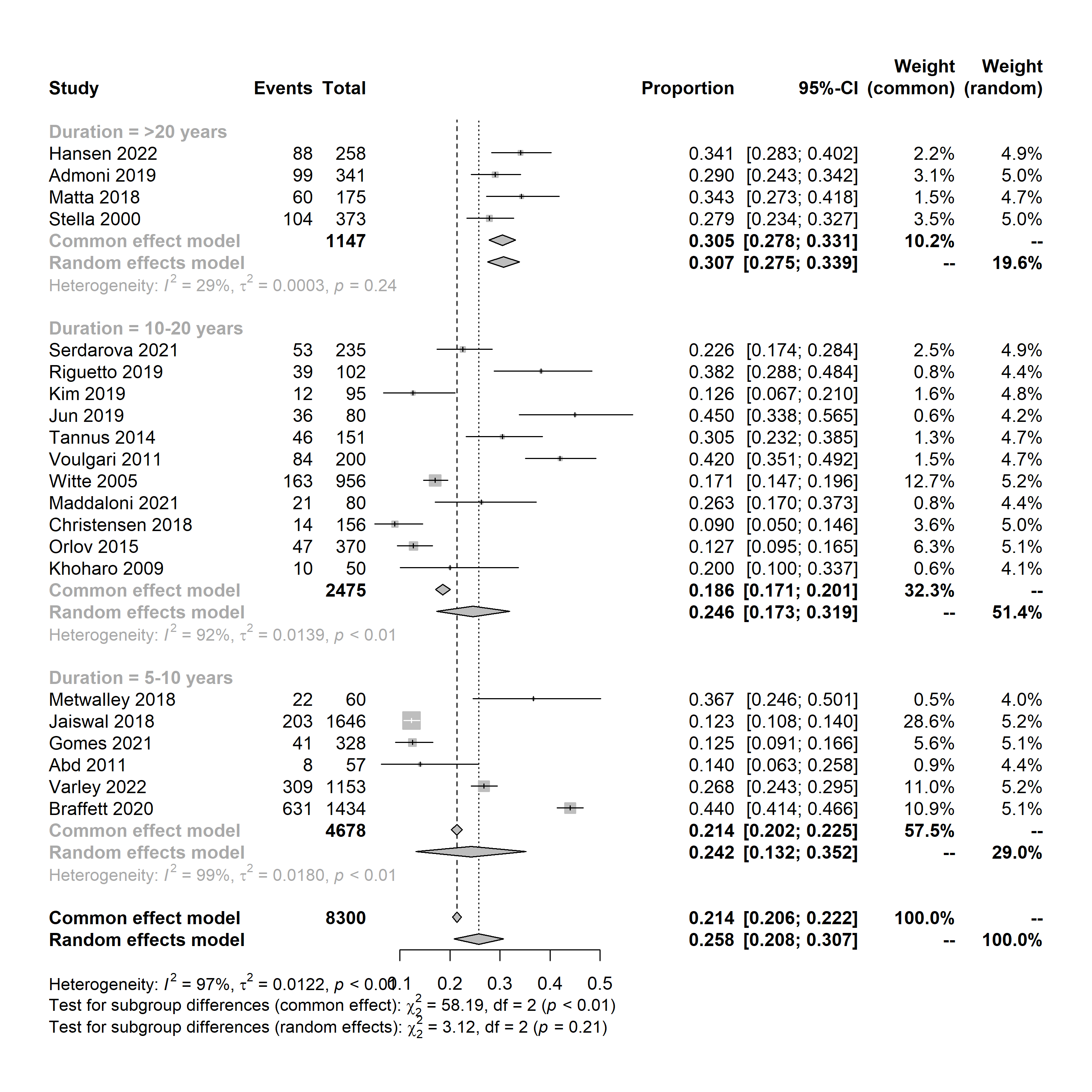


**Fig. 5. Subgroup analysis of prevalence according to** **diabetes duration.**


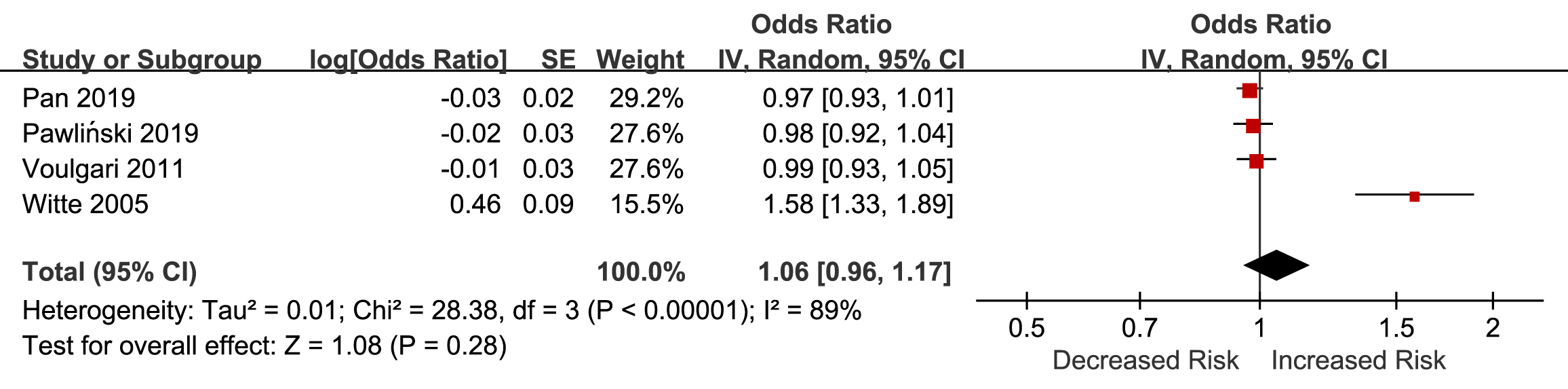


**Fig. 6. Forest plot for meta-analysis of age risk factors.**


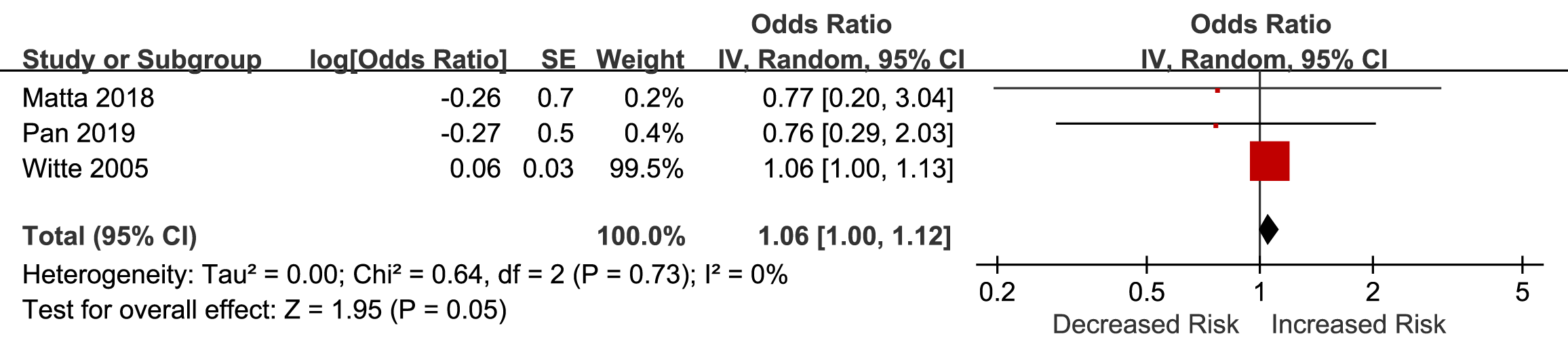


**Fig. 7. Forest plot for meta-analysis of BMI risk factors.**


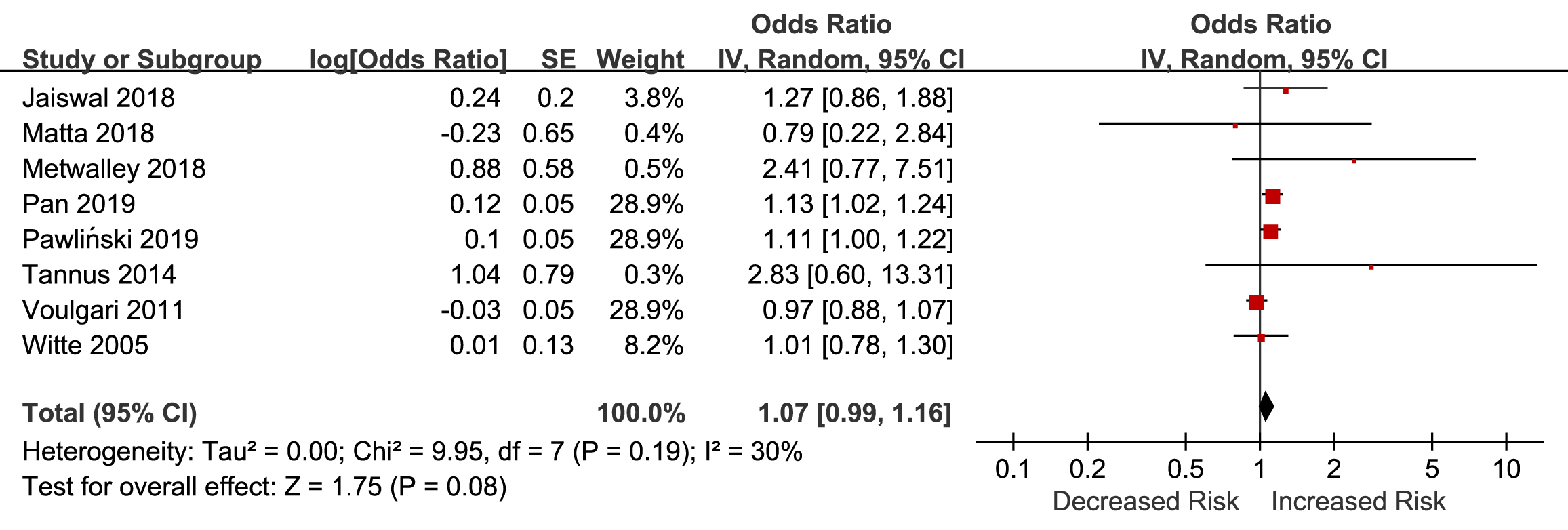


**Fig. 8. Forest plot for meta-analysis of Diabetes duration risk factors.**


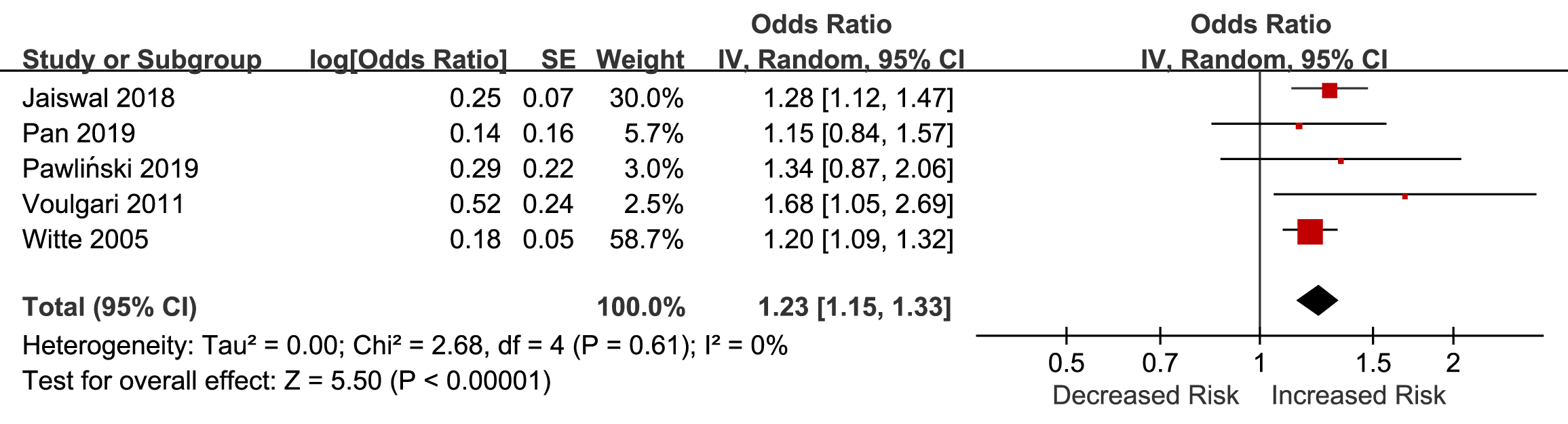


**Fig. 9. Forest plot for meta-analysis of HbA1c risk factors.**


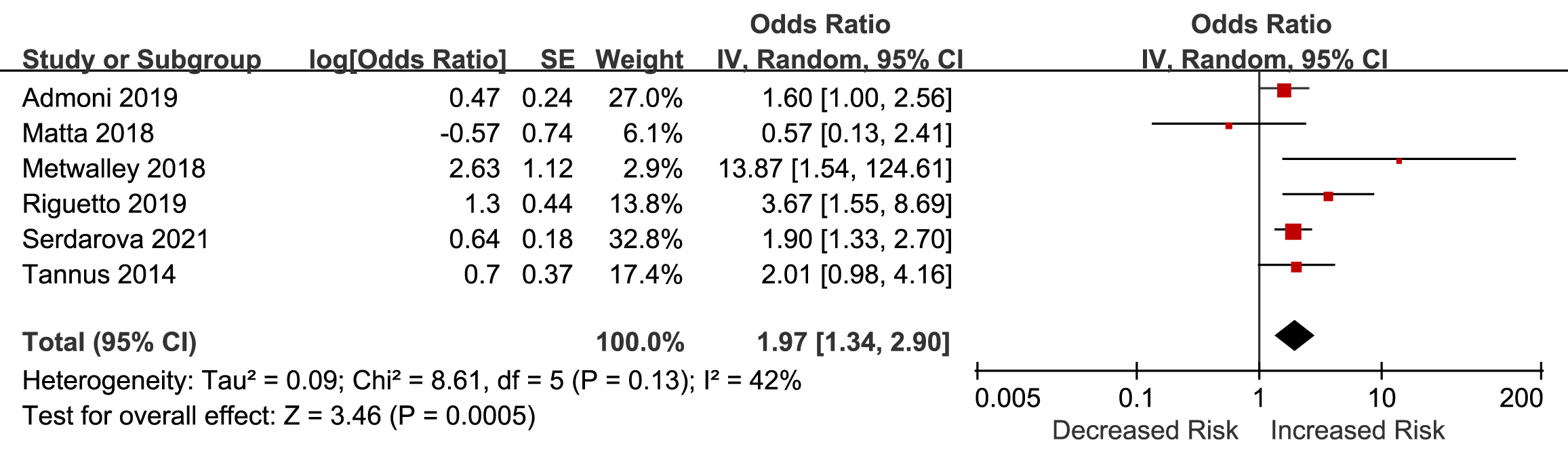


**Fig. 10. Forest plot for meta-analysis of Dyslipidemia risk factors.**


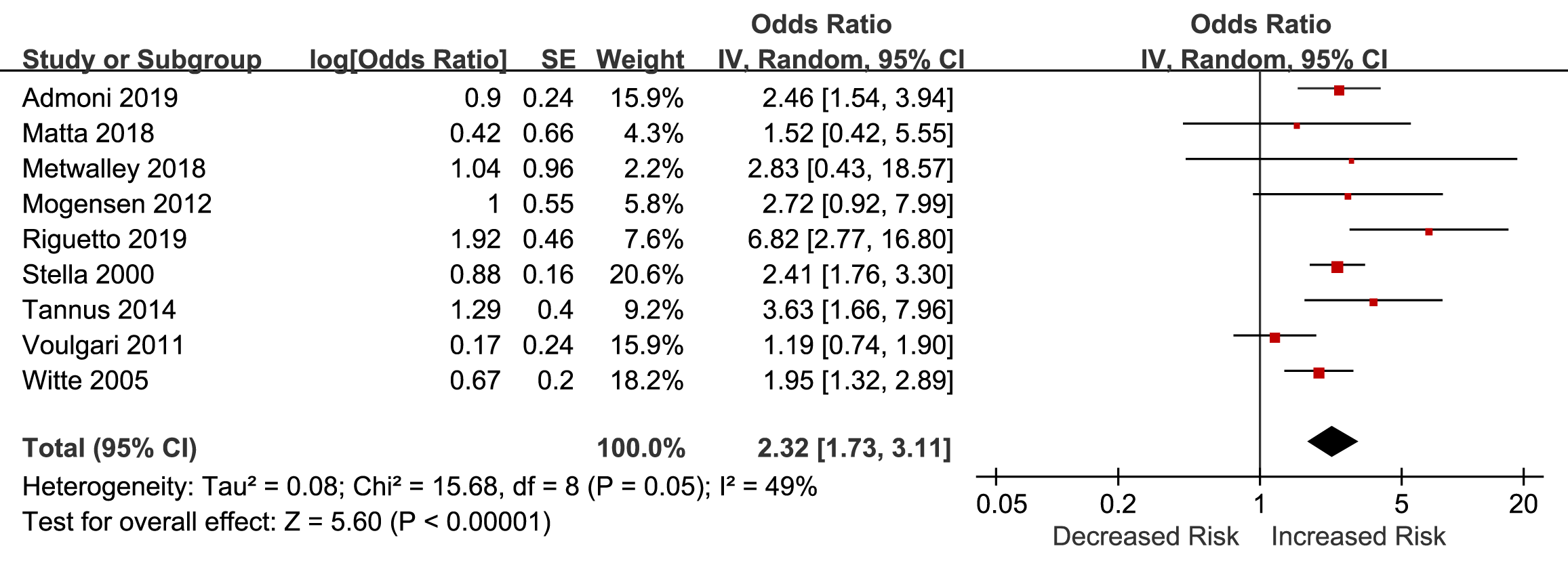


**Fig. 11. Forest plot for meta-analysis of Hypertension risk factors.**


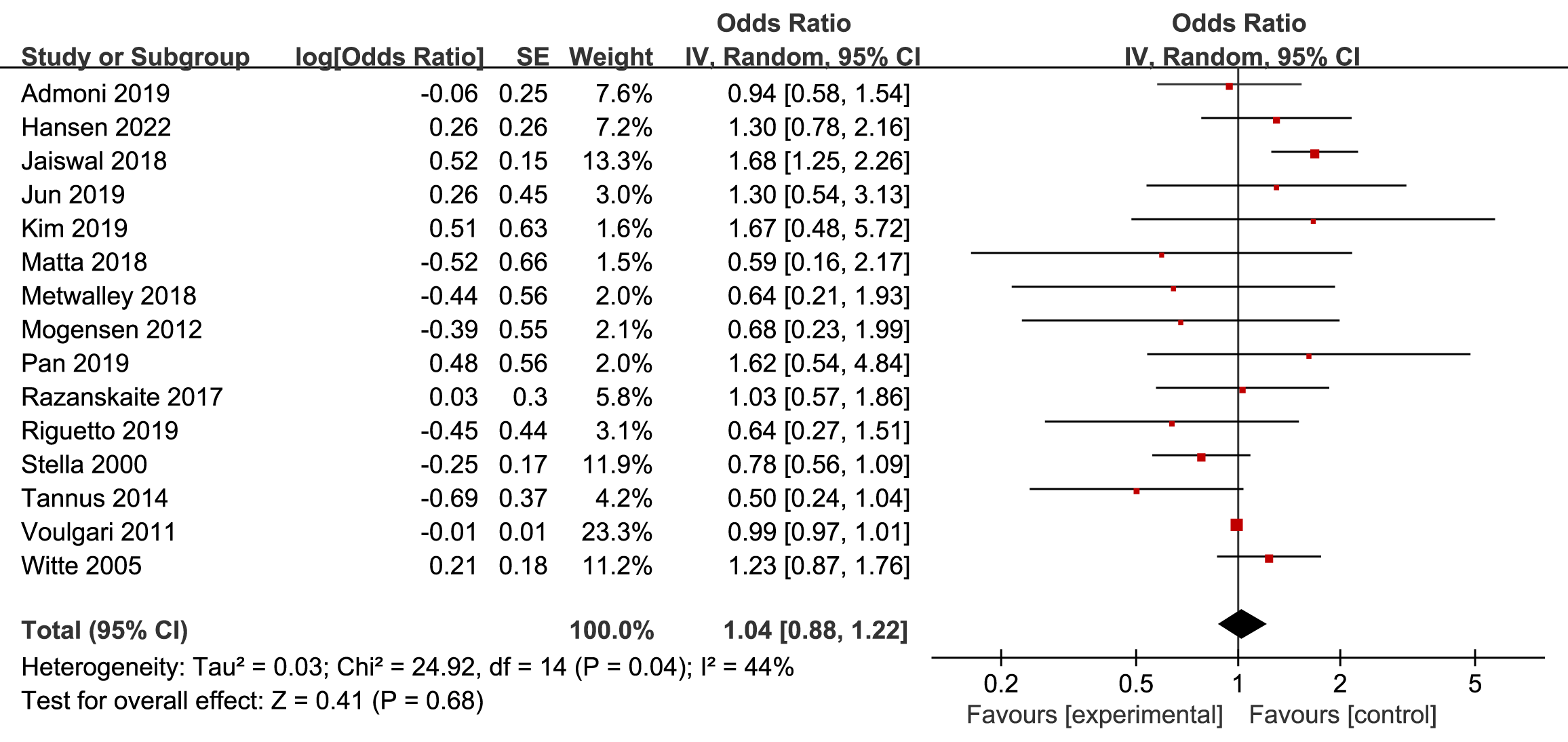


**Fi****g. 12. Forest plot for meta-analysis of Sex risk factors.**


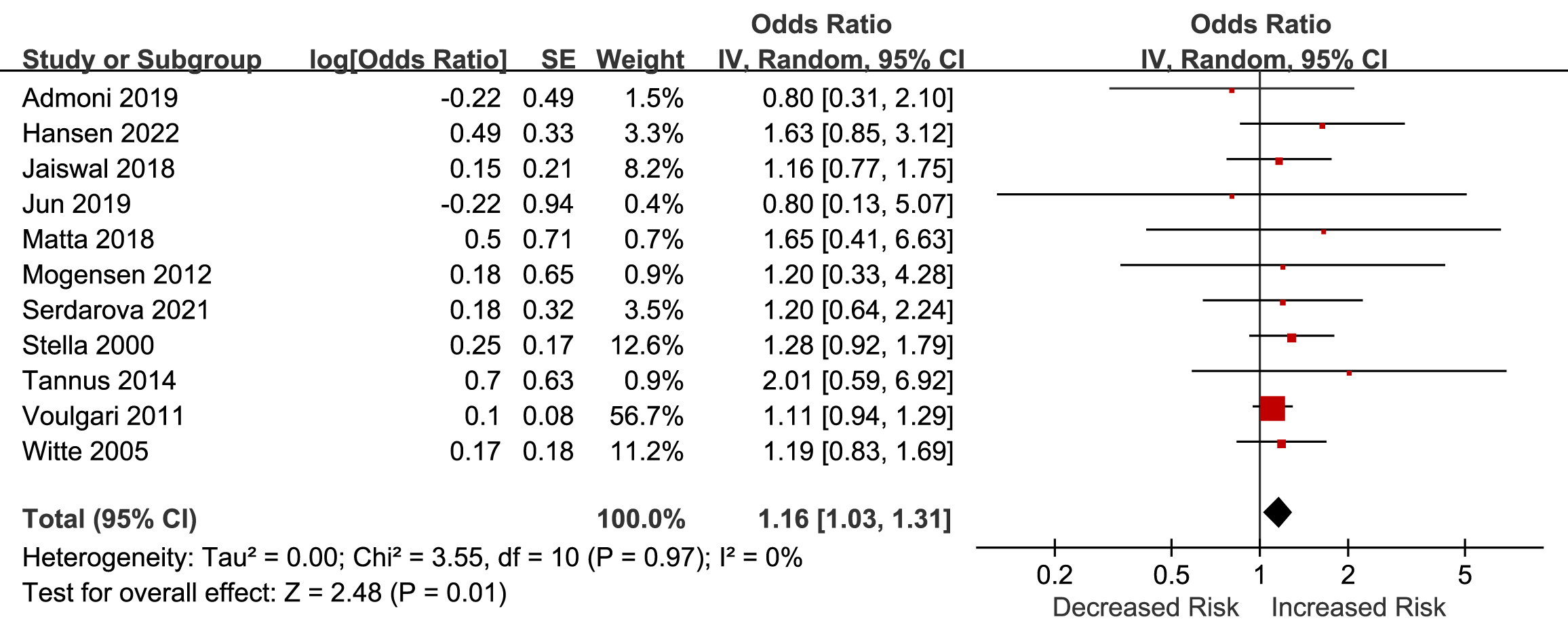


**Fig. 13. Forest plot for meta-analysis of Smoking risk factors.**


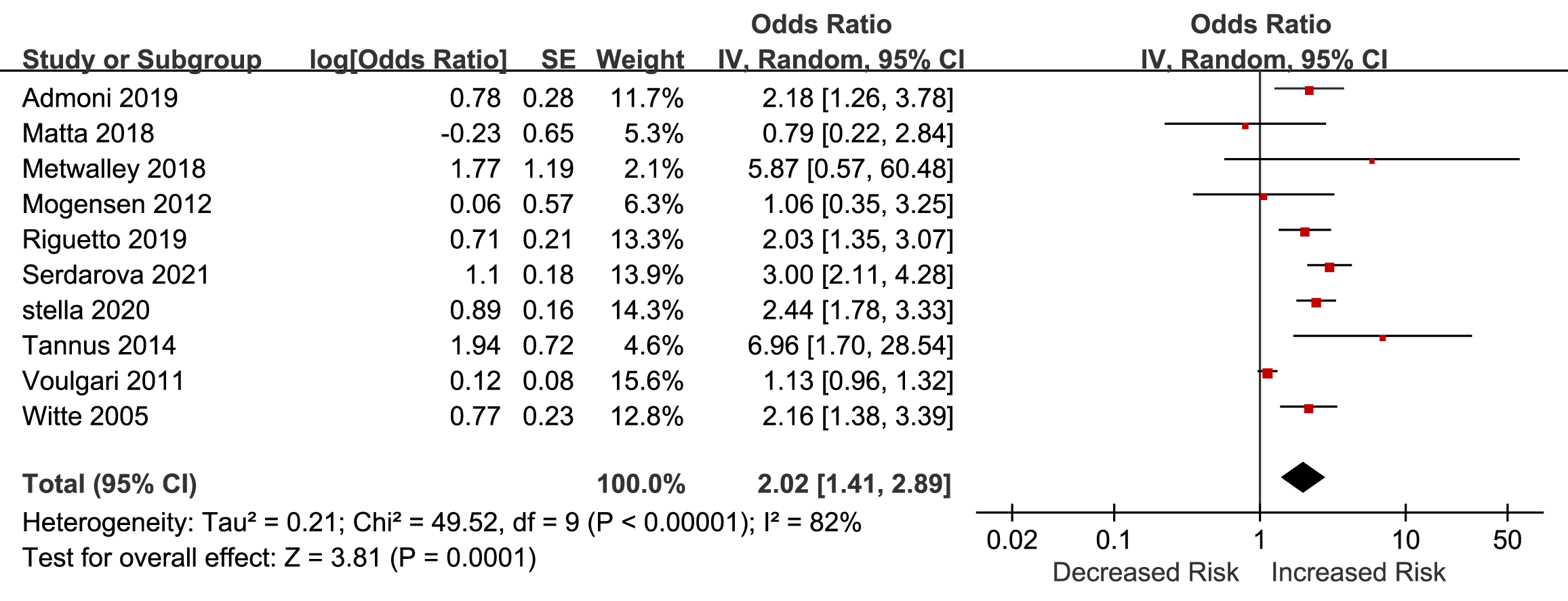
**Fig.14. Forest plot for meta-analysis of Diabetic Retinopathy risk factors.**


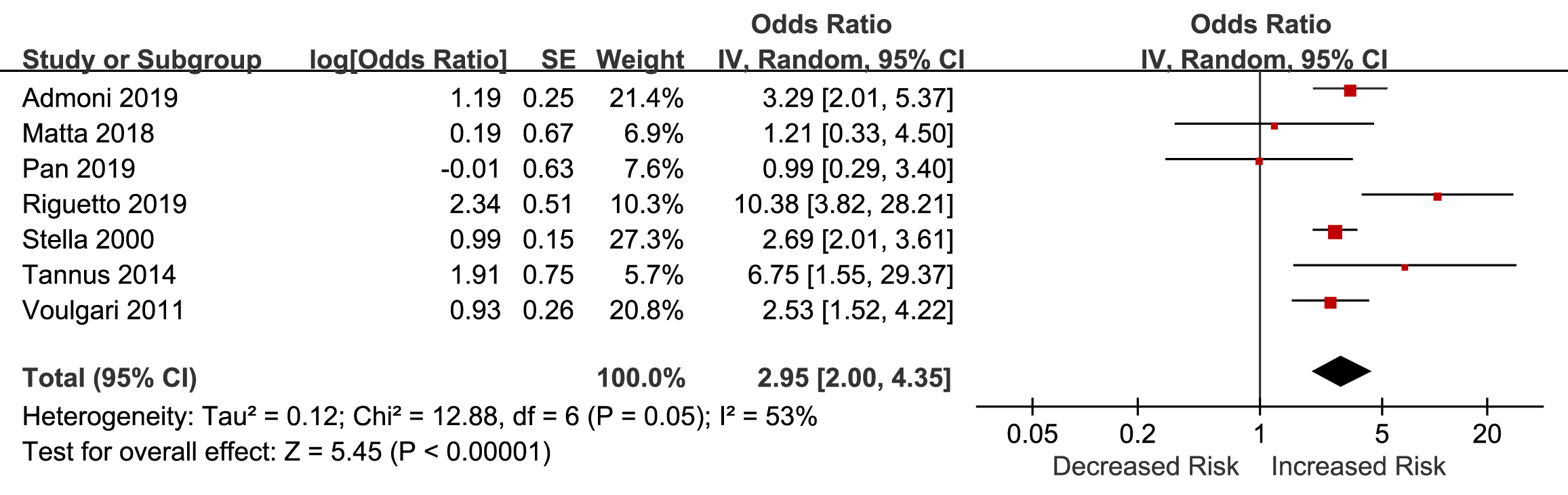


**Fig. 15. Forest plot for meta-analysis of** **Diabetic Reuropathy risk factors.**


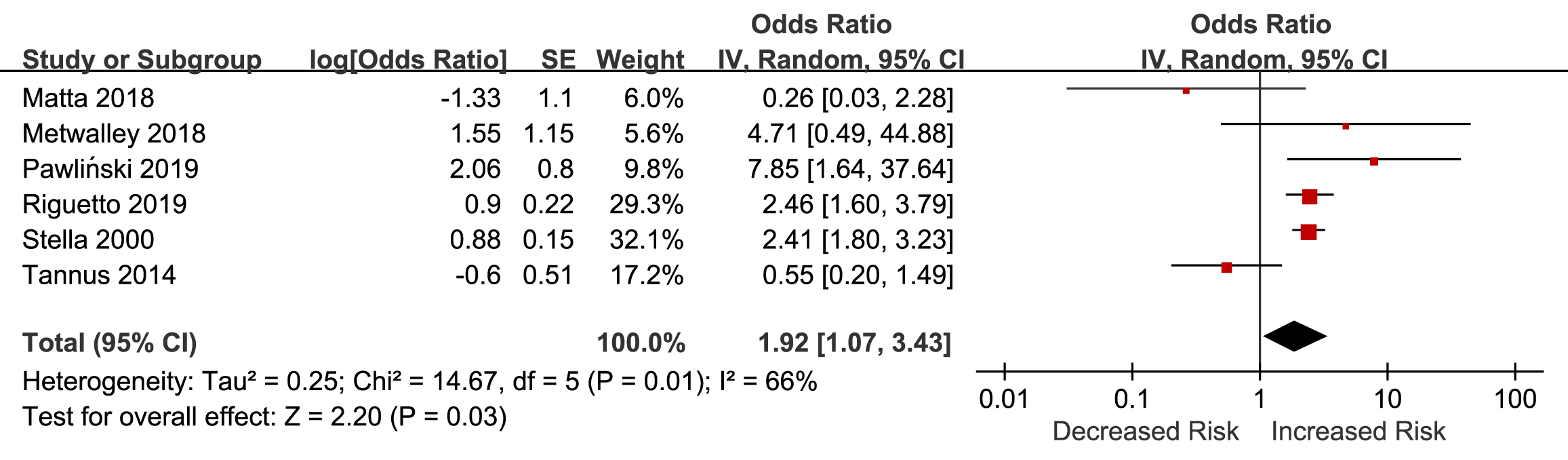


**Fig. 16. Forest plot for meta-analysis of** **Diabetic Nephropathy risk factor.**
